# Supplementary material for: The Impact of the SARS-CoV-2 Pandemic on the Needs of Non-Infected Patients and Their Families in Palliative Care—Interviews with Those Concerned
Source: J Clin Med. 2022 Jul 3;11(13):3863. doi: 10.3390/jcm11133863 (PMC9267922; doi:10.3390/jcm11133863)
Supplement: Supplementary file 1 [file jcm-11-03863-s001.zip › jcm-1749013-supplementary.pdf]

Journal of
Clinical Medicine

MDPI

Table S1. Sampling frame.

		Palliative Care Unit		Specialist Palliative Home Care	
		**Patients (5-6)**	**Family Caregivers (5-6)**	**Patients (5-6)**	**Family Caregivers (5-6)**
**Age**	Younger than 45 years	1-3	1-3	1-3	1-3
	Younger than 65 years	1-3	1-3	1-3	1-3
	65 years and older	1-3	1-3	1-3	1-3
**Gender**	Woman	2-3	2-3	2-3	2-3
	Man	2-3	2-3	2-3	2-3
**Family Status**	Single	2-3	2-3	2-3	2-3
	In a relationship	2-3	2-3	2-3	2-3

Table S2. Interview guide.

<table>
<tr><th colspan="3">Interview guide</th></tr>
<tr><td colspan="3">Impact of the current SARS-CoV-2 pandemic on the needs of patients without a SARS-CoV-2 diagnosis receiving palliative care and their families</td></tr>
<tr><td colspan="3">Before the interview</td></tr>
<tr><td>Participant information
Space for questions
Informed consent
Explaining the interview
(Continuous text for the ethics application – during the interview free explanation of the points mentioned here)</td><td colspan="2">Thank you very much for agreeing to participate in this study.
Before we begin, I'd like to briefly explain the goal of the study again.
These are turbulent times that we live in. We want to use the context of the current situation with COVID-19 to gain an understanding of patients receiving palliative care and their relatives that is as accurate and comprehensive as possible. That means we want to understand what is especially important to you as a patient / you as a relative at the moment and what your concerns or thoughts are.

I will ask some questions about your current situation in a moment. Please tell me about your own views and experiences about it. I am aware that you are in a special phase of life and there are many things around you that are developing dynamically and are changing. So, feel free to share anything you can think of – there are no wrong answers – your current views as well as those of the past weeks/months are welcome.
During the interview, we can always take a break if you want. Also, we can terminate the interview at any point. I will keep an audio recording running during the interview, so we do not lose anything said.
Do you have any questions about this?
Start of the recording.</td></tr>
<tr><td colspan="3">Fulfilment of needs</td></tr>
<tr><td>Main question</td><td>Adressed?</td><td>Potential follow-up questions</td></tr>
<tr><td>1. What is currently on your mind regarding your situation?
Alternatively: As a family caregiver, what is currently on your mind regarding your situation?</td><td>Current themes and needs</td><td>Could you elaborate on that?
What else can you think of?</td></tr>
<tr><td>2. Now, thinking back to the situation before COVID-19, a couple of months ago – that is quite a while:</td><td>Change regarding:
- Greater relevance
- Reduced relevance
- New topics</td><td>
Which topics became more important for you?
Which topics became less important for you?
Did any new issues arise? If yes, which?</td></tr>
</table>

How have the topics that concern you changed as a result of the COVID-19 situation?		
**Implementation of care**		
Main question	Adressed?	Potential follow-up questions
3a. What is your experience of the care you are receiving in this hospital / from the home care team?<br>Alternatively: How are you experiencing the care your mother, father, etc…. is receiving in this hospital / from the specialist palliative home care team?	Decision-making ability and sense of control<br>Communication<br>Relationship with doctors/nurses<br>Change	Which decisions did you have to make in the past few months? *…giving the participant opportunity to reply, then:* How did the COVID-19 situation influence these decisions?<br>What is your experience regarding the exchange of information / the communication with the treatment team?<br>In your opinion, which consequences arose from the COVID-19 situation regarding the care?
3b. (additionally for family caregivers:)<br>How do you personally feel about that?		
**Description of fears or worries**		
Main question	Adressed?	Potential follow-up question
4. Which worries and fears are you particularly concerned about because of the COVID-19 situation?	Social isolation<br>Uncertainty/certainty<br>Preparations	Which protective measures and contact restrictions did you follow?<br>How did that make you feel? Or rather, which effect did that have on your emotional state?<br>How did/does the COVID-19 situation influence your attitude toward life? What plans have you been pursuing in the past months? How did the COVID-19 situation influence these plans?
5. What is helping you or giving you strength right now?	Internal resources/coping strategies<br>External resources	How do you support yourself in coping with the COVID-19 situation?<br>Which external sources of support for coping with the COVID-19 situation do you have?
**Conclusion; space for reflecting on the conversation and asking questions; saying good-bye**	Is there anything we did not cover that would be important for you in the context of this conversation?	
**Participant characteristics**	Participant characteristics:<br>Context of care:<br>Age:<br>Gender:<br>Marital status:	□ Patient □ Family caregiver<br>□ Palliative care unit □ Specialist palliative home care<br>___________ years<br>□ Female □ Male<br>□ Single □ In partnership or married

Table S3. Example quotes for pandemic-independent needs.

Main theme	Subthemes	Example quotes[1]	
		**Patients**	**Family caregivers**
**Needs concerning physical aspects**	Symptom control	"Well, I always have a dry mouth due to this radiation. And I had a phase where I had such back pain and nausea." (2_029; palliative care unit)	"Because he's nauseous, because he has to vomit and so on and so forth, right." (2_021; specialist palliative home care)
	Being physically disabled	"Yeah well, so what keeps me busy is that I (…) my whole day I spend here lying down. I can move my finger a bit here and my feet a bit and that's it." (2_013; specialist palliative home care)	"She can neither swallow nor she can speak." (2_002; palliative care unit)
	Phases of the progressive disease	"First of all, I was again not feeling so well, at the end of December I just had another surge of the active metastases in the bone system, right? So then from the beginning of January to mid-May I was irradiated from top to bottom." (2_015; specialist palliative home care)	"(...) And then [in the dying phase] she also has glitches in between. And today she took a deep breath." (2_002; palliative care unit)
**Needs concerning emotions**	Emotional distress	"YES, I also often have these low points where I do actually say to myself, what's the point? For me this actually is a life that (…) it's just not worth living" (2_013; specialist palliative home care)	
	Anxious feelings	"But I also don't want to suffer unnecessarily. I'm not afraid of DYING itself. I am rather scared that it could be painful (...)." (2_015; specialist palliative home care)	" Well, of course, what happens next, there are a lot of fears. I mean we're in a palliative ward here of course there are a hell of a lot of fears, you don't know how things will progress, what…of course that preoccupies you." (2_001; palliative care unit)
	Feelings of loss	"When you suddenly CAN'T do something that you used to enjoy doing, THAT does hurt." (2_005; palliative care unit)	"You know of course that my husband IS in a palliative situation. And of course I am preoccupied about what will happen when he at some point won't be THERE anymore. Eh? What will life look like for me? Or rather, how will I cope with the pain, the grief?" (2_021; specialist palliative home care)
	Gratitude	"I am the OLDEST of the whole family anyway. They all died at eighty, eh? So eighty is the cut-off point in our family. And I am	

		now already eighty-EIGHT. And I'm turning eighty-NINE in January, right? Right. You have to be grateful for that." (2_019; specialist palliative home care)	
**Needs concerning existential and spiritual aspects**	Spirituality	"For me it's done then and probably a relief, eh? Because I don't at night when sleeping (…) I mean peaceful fall asleep and not wake up anymore." (2_015; specialist palliative home care)	"Well that someone dies, definitely. Yes, I mean, you live to die. That's senseless. (Laughter) But somehow, but nothing stays, nothing is forecer in the universe. (…) Yes. (…) At some point it happens to all of us. (…) Nothing, yes, nothing is as certain as death. (Laughs)" (2_004; palliative care unit)
	Life review	"I have done EVERYTHING in my life. I have ticked everything off the list. And thus I don't miss anything." (2_005; palliative care unit)	"Well (…) I mean, (…) when you have a spouse or a life partner who is doing as poorly as my husband, then you of course rethink the (…) relationship you had. We have been together for fifty years now. (…) And my husband also said, (…) well, when he could still talk, he said: 'These fifty years were actually not bad.' I mean 'not bad' from my husbad's mouth is high praise, (laughs)." (2_008; palliative care unit)
	Inner peace	"Yeah, at the moment, as I said, I am lying here. It's coming to an end. Everything is in order and done. So there's actually nothing else there." (2_016; palliative care unit)	"(...) because she just can't let go of that, the AUNT [patient]." (2_002; palliative care unit)"
	Desire to die	"And actually, I don't want to live for that much longer." (2_013; specialist palliative home care)	"Because she [patient] also just says, she's made her peace with death. She knows, THAT it won't GET better. But that she now, well, she WAITS just more or less for death." (2_023; specialist palliative home care)
**Needs concerning social aspects**	Social relationships	"(...) actually, just my family, my wife above all, my children, my grandchildren. That is what gives me strength." (2_013; specialist palliative home care)	"(...) now it's about making it possible for her to leave comfortably and no longer about healing. And so I do everything I can to make it as pleasant as possible, am THERE every day." (2_011; palliative care unit)
	Family caregiver burden	"(…) because of my illness, there are already enough people suffering, especially my wife of course, and what is important for me is that she suffers as little as possible from my last step." (2_003; palliative care unit)	"In everyday life, as a relative who also provides care at home, I am always busy ensuring this 24-hour care. (…) It is like a care ward. Sometimes like an intensive care ward, right? And that's not just something where you go to the old people's home for a

			visit, but that's the permanent life context, right? Before work, after work, at the end of the day. Right? So I do feel burdened with this." (2_018; specialist palliative home care)
	Needs emerging from the individual biography	"[My] relationship with my stepdaughter is significantly closer than with my stepson. (…) Yes, he is very full of himself, so the relationship was never very special, I would say. And with my stepdaughter I had a great relationship right from the start, just like a father would always wish for a daughter ". (2_028; palliative care unit)	"And my girlfriend is having a baby soon. I myself had an operation about a month ago. (…) Which in ITSELF is now encompassing such a healing process." (2_004; palliative care unit)
**Needs concerning coping with the disease**	Acceptance of the palliative condition	"And because of that, (…) others would despair now and freak out. Well, I've accepted it, you know?" (2_015; specialist palliative home care)	"And THEN, simply, yes, the realisation and the understanding by now, that there is in fact no healing for my mother anymore and ultimately the intention has changes. So away from a possible cure, because that is illusory, that will no longer happen, towards making the last phase of life as pleasant as possible." (2_011; palliative care unit)
	Prognostic awareness and hope	"Because, as I said, nobody can tell me how much longer." (2_015; specialist palliative home care)	
	Changes in goals of care and treatment decisions	"I thought that when I stopped chemo I would somehow fall into a hole. And that's exactly what didn't happen. (...) That I somehow still have a container for coping." (2_005; palliative care unit)	"But eventually it is the only right decision, I think. A cure is just not possible anymore." (2_011; palliative care unit)
	Adjustment to stages of the illness trajectory	"And then you start thinking and then you say: 'Yeah well, you are in a palliative situation.'" (2_015; specialist palliative home care)	"So I was in the clinic today. And I have to say that it would go so QUICKLY, that he so quickly (…) Yes, (…) yes, that is a shock in a way. Yes." (2_002; palliative care unit)
**Needs concerning autonomy**	Self-determination and choice	"I still HAVE [a] bit of independence. And I'm doing well with it. And more than that I don't want." (2_005; palliative care unit)	" And the thought of maybe going back to hospital and having chemo as an infusion, well, he said right away, there's no way he's going to do that." (2_021; specialist palliative home care)
	Dependency	"And I often experience myself also as a burden for the others." (2_013; specialist palliative home care)	

	Limitations of activities in everyday life	"And, yes, simply this time, how do I pass it best, how do I SPEND this time? And, yes, there's a lot going through my head." (2_013; specialist palliative home care)	"And we are very happy about our garden, eh? Which we really enjoy and take pleasure in. Eh? That we can sit outside for a bit and say about the rose: 'Oh, it's so beautiful.'" (2_021; specialist palliative home care)
**Needs concerning delivery/quality of care**	Issues of accessing and receiving care in the past	"Yeah, I have to say (…) the doctors, there are such differences. You wouldn't believe it. ONLY when you get to know a few others (…) then you just notice that in one practice you're just a number. Yeah? Nothing more. " (2_019; specialist palliative home care)	"Because she told me about these scenes (…) where some kind of examinations were due, and then a carer came and basically took her to the basement, and there was an appointment, and they weren't ready yet, and then she just stood outside in the corridor for half an hour." (2_011; palliative care unit)
	Perceptions of palliative care quality	"So, this is now my second day [on the palliative care unit] and (…) I couldn't wish for it to be any better." (2_006; palliative care unit)	"That's why we were so relieved actually about this palliative service, which then tried to regulate him right away with the painkillers." (2_021; specialist palliative home care)
	Next steps in palliative care	"Well, the important thing for me right now is that I'm going to the hospice on Friday. And yes. That's the next appointment coming up for me. And after that, I'll see what happens. Organising this now, so that my wife can cope with it, too." (2_028; palliative care unit)	And I really hope for her that we will be able to make this way to the hospice, (…) because they are incredibly caring and nice and really ACCOMPANY you with this end-of-life care." (2_011; palliative care unit)
	Security	"I KNOW when something is UP or when I have problems, then I know, where to call and then I will get help. (…) I just feel safe because of that, (…) And if there really is an emergency, then I also know that they will react quickly. And that simply reassures me. And that is the main think, I have to say, for me." (2_013; specialist palliative home care)	"Well, this is really great, that they are always there to support you, no matter WHEN you need them. So we, thank God, have NOT had the emergency yet, but we always write down our questions for the next conversation or something, right? If it's nothing urgent, then we of course don't want to bug them. They probably have more URGENT cases anyway, than we are. But it is a good feeling at least, to know, yes, there's someone you can call at all times, right?" (2_021; specialist palliative home care)
**Needs concerning preparations for death**	Bureaucracy including setting up powers of attorney	"But this way we HAVE already sorted many things so that she has little work with it afterwards, right? She'll be stressed then anyway. She'll have to deregister certain things, life insurance	"Everything that makes up normal everyday life, so to speak, and the sea of bureaucracy, I'll say, be it health insurance, pension insurance, ah, all such things, too, which are now kind of up to me and which I now have to tackle and also to deal with, so to speak,

		AND so on and so forth." (2_015; specialist palliative home care)	and that is simply a huge balancing act. Which now also goes on for a long time, (…)." (2_030; palliative care unit)
	Planning for funeral arrangements		"what the funeral home does then, what we have to do OURSELVES, what kind of DRESS I have to pick out for my mother, (...) who we invite to the funeral service, that kind of thing." (2_009; specialist palliative home care)

[1] Blank fields mean no mention in the interviews. It should be in mind that the explored topics were not queried deductively.

S4 Membership of the PallPan Study Group

PallPan is a network of 13 university palliative care institutions in Germany. It cooperates closely with other institutions and people who are active in research in the field of palliative medicine and hospice work. The research association was founded within the framework of the Network University Medicine (NUM) and funded by the Federal Ministry of Education and Research (BMBF). In the Corona Pandemic 2020/2021, the PallPan consortium has implemented several research projects on the topic of "Palliative Care & Hospice Work in a Pandemic" with the aim of recording burdens, problems and possible solutions. The main goal is the development and continuous updating of a national strategy for the care of severely ill, dying, and deceased adults and their relatives in times of a pandemic in Germany, which was published for the first time in June 2021. Recommendations for action and information material on palliative care for patients with/without infection were jointly developed for all care sectors that treat and accompany severely ill and dying people and their relatives.

Homepage: https://pallpan.de/uber-pallpan/

Membership:

Department of Palliative Medicine, Ludwig-Maximilians-University Munich, Munich University Hospital, Munich, Germany.

Palliative Care Unit, Department of Oncology, Hematology and Bone Marrow Transplant, University Medical Center Hamburg-Eppendorf, Hamburg, Germany.

Department of Palliative Medicine, University of Cologne, Faculty of Medicine and University Hospital, Cologne, Germany.

Hannover Medical School, Institute for General Practice and Palliative Medicine, Palliative Care Research Group, Hannover, Germany.

Department of Palliative Medicine, University Hospital Aachen, Aachen, Germany.

Department of Palliative Medicine, Jena University Hospital, Jena, Germany.

Department for Palliative Medicine, Georg August University Goettingen, University Medical Center, Goettingen, Germany.

Interdisciplinary Center for Palliative Medicine, University Wuerzburg, University Hospital, Wuerzburg, Germany.

Interdisciplinary Center for Palliative Care, Heinrich Heine University Duesseldorf, University Hospital, Duesseldorf, Germany.

Department of Anesthesiology, Heinrich Heine University Duesseldorf, University Hospital, Duesseldorf, Germany.

Department of Palliative Medicine, University Hospital Bonn, Bonn, Germany.

Department of Palliative Medicine, Comprehensive Cancer Center, CCC Erlangen – EMN, Friedrich-AlexanderUniversität Erlangen-Nürnberg (FAU), University Hospital Erlangen, Erlangen, Germany.

Department of Medicine, Clinic III-Hematology, Oncology, Palliative Medicine - CCC Mecklenburg-Vorpommern, Rostock University Medical Center, Rostock, Germany.

Faculty of Medicine, Medical Center, Department of Palliative Medicine, University of Freiburg, Freiburg, Germany.

Faculty of Medicine and University Hospital, Department I of Internal Medicine, University of Cologne, Cologne, Germany.

Faculty of Medicine and University Hospital, Center for Integrated Oncology Aachen Bonn Cologne Dusseldorf (CIO ABCD), University of Cologne, Cologne, Germany.

Faculty of Medicine and University Hospital, Clinical Trials Center (ZKS), University of Cologne, Cologne, Germany.

Faculty of Medicine and University Hospital, Center for Health Services Research, University of Cologne, Cologne, Germany.
